# A honey bee (*Apis mellifera *L.) PeptideAtlas crossing castes and tissues

**DOI:** 10.1186/1471-2164-12-290

**Published:** 2011-06-03

**Authors:** Queenie WT Chan, Robert Parker, Zhi Sun, Eric W Deutsch, Leonard J Foster

**Affiliations:** 1 Centre for High-Throughput Biology and Department of Biochemistry & Molecular Biology, University of British Columbia, Vancouver, Canada; 2 Institute for Systems Biology, Seattle, WA, USA

## Abstract

**Background:**

Honey bees are a mainstay of agriculture, contributing billions of dollars through their pollination activities. Bees have been a model system for sociality and group behavior for decades but only recently have molecular techniques been brought to study this fascinating and valuable organism. With the release of the first draft of its genome in 2006, proteomics of bees became feasible and over the past five years we have amassed in excess of 5E+6 MS/MS spectra. The lack of a consolidated platform to organize this massive resource hampers our ability, and that of others, to mine the information to its maximum potential.

**Results:**

Here we introduce the Honey Bee PeptideAtlas, a web-based resource for visualizing mass spectrometry data across experiments, providing protein descriptions and Gene Ontology annotations where possible. We anticipate that this will be helpful in planning proteomics experiments, especially in the selection of transitions for selected reaction monitoring. Through a proteogenomics effort, we have used MS/MS data to anchor the annotation of previously undescribed genes and to re-annotate previous gene models in order to improve the current genome annotation.

**Conclusions:**

The Honey Bee PeptideAtlas will contribute to the efficiency of bee proteomics and accelerate our understanding of this species. This publicly accessible and interactive database is an important framework for the current and future analysis of mass spectrometry data.

## Background

The honey bee, *Apis mellifera *L., best known for its honey production and pollination of crops, has been making headlines in the past half-decade. Under increasing threat from disease and chemical residues in the environment, research efforts on this beneficial insect have escalated in the past five years. After completion of the honey bee genome sequence in 2006 [1], the next step is to understand the proteome. Implicit in this goal is that the expressed proteome of the bee must first be defined: bioinformatic analyses place the number of honey bee genes near 10,000, (summarized in [2]) but only a handful of these have been observed experimentally at the protein level. Since most gene prediction algorithms require a training set of genes with well-established translation start/stop sites and intron-exon boundaries, the lack of even a hundred bee genes with real experimental evidence, let alone annotated to this level of quality, hinders prediction efforts. Homology-based methods using *Drosophila *and other organisms with well-annotated gene lists have helped to find core bee genes, yet the relatively large evolutionary distance between *Drosophila *and *Apis *(~300 million years) poses limitations on this approach. For example, the eusociality of bees implies the expression of genes that flies, being solitary insects, would not have. mRNA sequences, such as from EST libraries, can help to map transcribed genes but experimentally verified proteins are still the ultimate affirmation of gene expression.

The emerging field of proteogenomics [3,4] applies the power of mass spectrometry proteomics to improve the genomic understanding for a species. Typically large proteomics datasets are processed using either a large set of ORF overpredictions or using the entire genome itself in order to identify sequences that are translated to protein but do not yet appear in annotated protein lists. This technique is computationally expensive, but a complete protein parts list is a key component in designing experiments to expand the understanding of a species.

In the last four years a plethora of bee proteomics studies have been published (summarized in [5]) but there is, as yet, no central resource dedicated to integrating all this data. Our group alone has acquired in excess of 5.5 million tandem mass spectra from bee samples, representing a rich source of data with which to validate many bee genes and possibly correct many of the annotations. The power of this approach has been demonstrated for the closest model organism, *Drosophila *[6].

The PeptideAtlas [7] provides a central, stable resource for mass spectrometry data supporting protein identification information for several species. Raw MS/MS data are processed through a single processing pipeline of sequence searching and post-processing with the Trans-Proteomic Pipeline [8,9] to yield a high quality list of identifications with a low and well-characterized false discovery rate (FDR). The resulting list is mapped onto the genome in order to provide chromosomal coordinates for all peptides. PeptideAtlas makes the results of this processing available to the community in a variety of ways, including a browser-based interface for viewing and querying the data [10]. Further, PeptideAtlas enables intra- and inter-species comparisons whose value is further increased by the application of a uniform analytic process across all species supported.

Here we present the following resources to the community in order to accelerate honey bee research of all kinds: we describe the building of the Honey Bee PeptideAtlas, a compendium of protein identification information derived from a large set of MS/MS data; we present a set of corrections to the honey bee proteome, a set of functional annotations based on the Gene Ontology classification based on homology to other species, as well as a comprehensive spectral library and a resource to enable emerging targeted proteomics workflows.

## Implementation

### Raw data collection

All the MS data were collected using either an LTQ-OrbitrapXL or LTQ-FT between 2005 and 2009. Samples included various organs and life stages, treatment and infection states, and strains selected for various traits such as disease resistance or pollen hoarding summarized in Table 1, all from the European honey bee *A. mellifera*. Some of the datasets have been published previously [5,11-13]. In solution and in gel digestion of samples was performed as described [12] and some were subsequently isotopically labeled to measure relative quantities between different conditions/tissues [5,11,13]. Highly concentrated samples were fractionated by strong cation exchange chromatography in a step gradient [14]; all samples were desalted using STAGE Tips [15] before injected into a nanoflow liquid chromatography system with C_18 _reversed phase material and sprayed into the mass spectrometer as described in [5].

**Table 1:** Honey bee castes and tissue samples in PeptideAtlas

Tissue	Caste	Developmental Stage	Number of RAW files
Whole	Indeterminate	egg	6

Hemolymph	Worker	larval instars 1-5	468

Solid tissue	Worker	larval instars 1-5	132

Brain	all	adult	85

Crop (foregut)	all	adult	16

Eye	all	adult	9

Galea	all	adult	13

Hemolymph	all	adult	122

Intestine	all	adult	21

Leg (front)	all	adult	39

Leg (mid)	all	adult	39

Leg (rear)	all	adult	34

Mandibular gland	all	adult	12

Mouth part	all	adult	1

Muscle	all	adult	11

Ocellus	all	adult	4

Rectum (hindgut)	all	adult	29

Salivary gland (post-cerebral)	all	adult	4

Salivary gland (thoracic)	all	adult	22

Ventriculus (midgut)	all	adult	36

Mucus gland	Drone	adult	33

Testis	Drone	adult	33

Spermatheca	Queen	adult	33

Abdomen	Worker	adult	82

Antenna	Worker	adult	7

Fat body	Worker	adult	1

Ovary	Worker	adult	1

Salivary Gland	Worker	adult	1

Thorax	Worker	adult	6

Wing	Worker, Drone	adult	16

Poison sac	Worker, Queen	adult	8

Sternite	Worker, Queen	adult	12

Tergite	Worker, Queen	adult	6

Pollen	-	-	3

### Correction and identification of new proteins

In order to detect possible new genes and proteins, as well as to correct incorrect protein predictions, all MS/MS spectra described above were searched using an automated pipeline built using Proteus (Genologics, http://www.genologics.com/). The pipeline automates submission of MS/MS spectra to an off-site Mascot server http://www.matrixscience.com/ and the retrieval and filtering of search results. The peak lists were initially searched against the NCBI *A. mellifera *protein database (plus human contaminants and digestion enzymes) using a Mascot score cutoff of 27, essentially as described [5]. Spectra that did not match any peptides from this search were re-searched against a six-frame translation of the honey bee genome. The six-frame translation was created independently of the built-in function in Mascot, using the eukaryotic genetic code and limiting an open reading frame (ORF) to at least 35 amino acids and spectral hits against this database were considered further if they had an IonsScore of at least 25. For a genomic six-frame translation library this cut-off is not very stringent but this was used simply as an initial filtering step. ORFs that were hit by at least two unique peptides were examined further to see if they could be missed exons of previously annotated genes or if they occurred far from any known genes and thus might be novel genes. ORFs meeting these criteria were then shortened to cover only the region spanned by peptide identifications and added to the protein sequence library used in PeptideAtlas. Links to all of these sequences can be found in Additional File 1, as well as at the honey bee download area at PeptideAtlas at http://www.peptideatlas.org/builds/honeybee/.

### Creation of a comprehensive protein set

In order to process the MS/MS data within PeptideAtlas against the widest array of possible honey bee proteins, we created a comprehensive protein set by assembling the Refseq ftp://ftp.ncbi.nih.gov/genomes/Apis_mellifera/protein/protein.fa.gz sequences, Official Gene Set 1 [1,16], Genbank [17], and Gnomon predictions ftp://ftp.ncbi.nih.gov/genomes/Apis_mellifera/protein/Gnomon_prot.fsa.gz. The three protein sets were merged by removing all exact duplicates and keeping the first of the protein in order of the sources as listed above. This was then supplemented with the new protein sequences described in the previous section. Note that only exact duplicates are removed, and many near duplicates remain as it is difficult to discern which are the result of sequencing errors from real SNPs or gene duplications. This new protein list may be downloaded at the PeptideAtlas honey bee download area.

### Construction of PeptideAtlas

All raw data were converted to mzML [18] using the msconvert tool from Proteowizard [19] bundled in the TPP [20]. The mzML files were searched using the protein set described above to which a shuffled decoy set had been appended. The sequences are shuffled by scrambling all amino acids between fixed tryptic cleavage sites. The data were searched with X!Tandem [21] with the K-score plugin [22]. X!Tandem output was processed with the TPP versions of PeptideProphet [23], iProphet (Shteynberg D, Deutsch EW, Lam H, Eng J, Sun Z, Tasman N, Mendoza L, Moritz RL, Aebersold R, Nesvizhskii AI: iProphet: improved statistical validation of peptide identifications in shotgun proteomics, submitted), and ProteinProphet [24] to extract the maximal identifications with highest confidence scores. All identifications were filtered at a peptide-spectrum-match (PSM) FDR threshold of 0.0001, which yielded a peptide-level FDR of 0.0018 and protein-level FDR of 0.015 as estimated using the decoy identifications. These filtered results were loaded into the PeptideAtlas database as build "Honeybee 2010-03" and can be downloaded and browsed in the usual manner [10].

### Predicting protein function with BLAST2GO

Since functional annotations are very incomplete for honey bee, we have attempted to greatly expand the protein annotations using the Gene Ontology (GO). The GO system organizes all protein annotations into a hierarchical structure of increasing granularity, with three separated root categories: molecular function, biological process, and cellular component. All proteins were processing through the BLAST2GO program [25] which yields a set of GO annotations for each protein based on homology to proteins from other species as determined by BLAST [26]. We used this software according to the default protocols and settings: BLAST searches were conducted for each protein (BLASTp, nr database, HSP cutoff length 33, report 20 hits, maximum e-Value 1e-10), followed by mapping and annotation (e-Value hit filter 1e-10, annotation cutoff 55, GO weight 5, HSP-hit coverage cutoff 20). The results of the BLAST2GO mapping may be downloaded at the same URL as previously provided for other data products described above.

## Results

**Figure 1:**
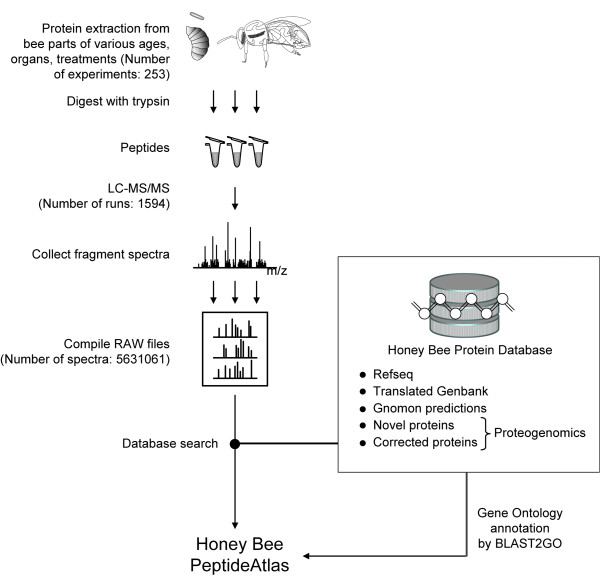
**Creating the Honey Bee PeptideAtlas**. Honey bee tissues of different ages, sexes, and disease states were analyzed by MS using the high accuracy LTQ-OrbitrapXL or LTQ-FT. To improve proteome depth, we used samples which were biological replicates (i.e. same experiment on more than one bee), technical replicates (i.e. one bee sample analyzed more than once by MS), and employed various fractionation techniques. The RAW files were put through the PeptideAtlas pipeline. The database used for searching the MS data contained sequences from various publicly available sources, as well as novel and corrected proteins derived from the proteogenomics effort reported in this article. Using BLAST2GO, GO terms were annotated to proteins where appropriate. This result of this work comprises the Honey Bee PeptideAtlas.

The Honey Bee PeptideAtlas (HBPA) was assembled from MS/MS collected over four years from all three castes, larvae and virtually all adult honey bee tissues as described in Methods and as outlined in Figure 1. The data were searched in a first-pass genomic search to identify a set of putative new protein sequences. All honey bee proteins were annotated via BLAST2GO. All MS/MS data along with the new annotation information were put through the PeptideAtlas pipeline to create the final product. The HBPA build resulted in over 1.3 million peptide-spectrum matches (PSMs) at a FDR of 0.0001. This results in 27,776 distinct peptide sequences at an FDR of 0.0018, mapping to approximately 3000 highly non-redundant proteins at a protein FDR of 0.015. The process of how the peptides are mapped to proteins and the proteins classified is described in detail elsewhere [27]. Further build statistics are listed in Table 2.

**Table 2:** Summary of the Honey Bee PeptideAtlas 2010-03 build

Build	Honey Bee PeptideAtlas 2010-03
Total Experiments	253

Total ms runs	1,594

Spectra searched	5,601,751

PSMs above threshold	1,339,806

Distinct peptides	27,776

Distinct proteins	3,009

Mayu Analysis	

PSM	TP_PSM: 1,339,586 FP_PSM 110 FDR: 0.0000821

Peptide	TP_pepID: 27,725, FP_pepID 51 FDR 0.00180

Protein	TP_protID: 3009, FP_protID 50 FDR: 0.0154

**Figure 2:**
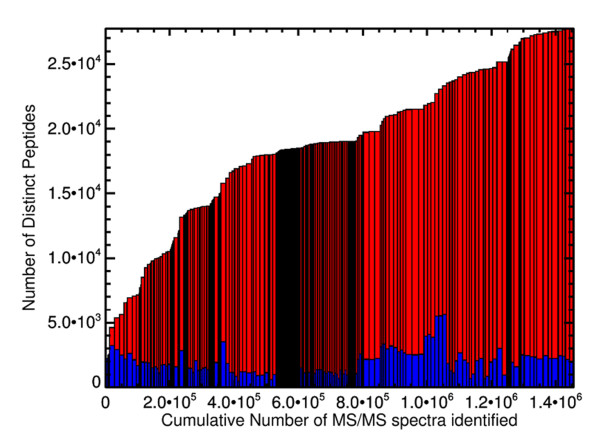
**Plot showing the cumulative number of distinct peptides added to the Honey Bee PeptideAtlas versus the total number of peptide-spectrum matches (PSMs) about the 0.0001 FDR threshold**. Each rectangle represents one of the 253 samples in the atlas. The height of each bar is the cumulative number of distinct peptides a sample is added; the blue component is number of distinct peptides in each sample. The width of the bar denotes the number of PSMs identified above the threshold for each sample. Groups of similar samples cause the cumulative number of distinct peptides to level off, but as a new sample type is added, the number of additional distinct peptides added to the build increases significantly.

The sequential addition of the 253 individual samples is depicted in Figure 2. Although each individual experiment only contains a few thousand peptides (as depicted by the blue component), the total number of distinct peptides in the entire build continues to increase as more samples are added. The addition of groups of similar samples in succession leads to multiple instances where the total peptide count rises sharply and then flattens out as replicates are added.

**Figure 3:**
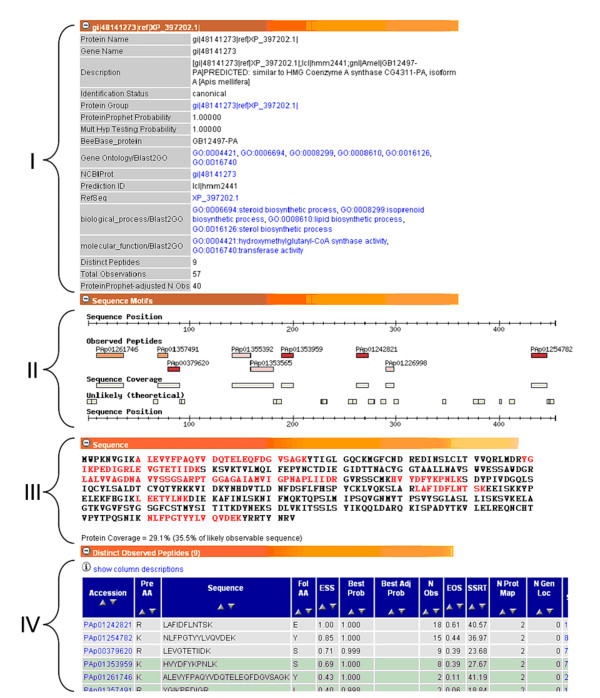
**Screenshot of a protein view within PeptideAtlas for protein GB12497-PA**. The general protein view has several collapsible sections that provide information about the protein. Section I provides known aliases and descriptions of the protein including functional annotations from our BLAST2GO results, while Section II depicts the distribution of observed and unlikely peptides in a graphical format. Section III shows the full amino acid sequence with observed parts colored in red. Section IV lists individual observed peptides and their attributes.

**Figure 4:**
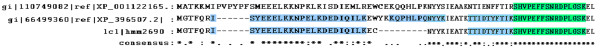
**Screenshot of a PeptideAtlas sequence alignment of three similar proteins**. The sequences are aligned with ClustalW, whose consensus string is show below the sequences; an asterisk indicates identity for all proteins. Sequence is colored blue or green where observed peptides are seen. There is no independent evidence that the top protein is detected, while there is significant evidence that the bottom form is detected.

The PeptideAtlas interface allows the user to explore individual proteins, where they map onto the genome and what MS/MS evidence supports their identity (Figure 3). It also allows one to compare the sequences of honey bee proteins to other bee proteins or even to those in other organisms supported by PeptideAtlas; in particular, peptide evidence for the presence of very closely related isoforms or family members can be displayed. The usefulness of this feature is exemplified in Figure 4: several similar variants of one protein have been predicted; a large amount of RAW data was searched, but MS/MS data clearly show support for only some of the variants and not others.

Honey bees are not a classic model system for molecular studies and as such, bee protein functions are relatively poorly annotated. Indeed, each time we have tried to analyze a proteomic dataset from bees [5,11,13], we have been forced to re-generate Gene Ontology (GO) [28] classifications for the proteins of interest. GO is a controlled vocabulary describing the molecular function, biological process and cellular component for gene products, where the same terms are used across all species. In order to provide an ontological classification scheme for bee proteins as an additional resource to the community, BLAST2GO [25] was used to assign tentative annotations to honey bee proteins based on the closest sequence homolog for which GO assignments are available. A total of 9009 sequences can be matched to at least one GO term, or about 37% of the sequences in the Honey Bee PeptideAtlas. The majority remains unmatched, primarily because the input sequences include ones from earlier annotations of the genome which have been eliminated in later versions; in searching our MS data against them we saw that some were falsely excluded since they match peptide spectra, however, we inevitably include protein sequences that are not actually translated. Another reason would be the lack of well-annotated insect proteins (e.g., relative to human and mouse) for bee proteins to match against. In performing manual checks, even highly abundant and important bee sequences such as major royal jelly proteins (the larval food source) and odorant binding proteins (soluble transporters of hydrophobic odor molecules) are not matched to any GO terms.

These assignments are integrated into the PeptideAtlas for each protein and hyperlinked to further information regarding the particular term. The annotations appear in the protein summary page for each protein, and one may search for all proteins associated with a given term via the main PeptideAtlas search page. For example, a search for "photoreceptor" yields 23 distinct proteins, which contained the query term either in the protein description or GO annotation. Each description is linked to a page pertaining to the protein of interest with further information regarding its MS evidence, frequency of observation, proteotypic peptides, and more.

**Figure 5:**
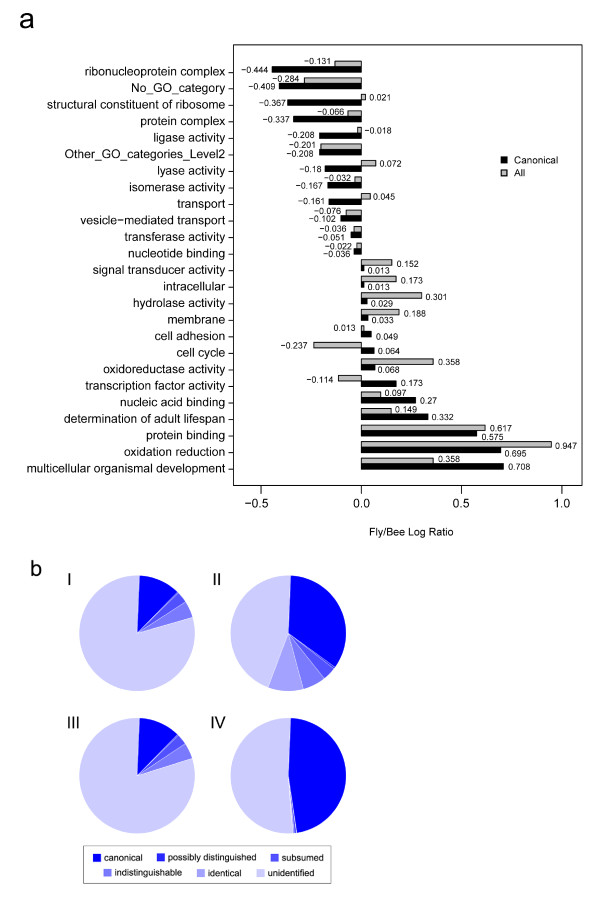
**Comparing the honey bee and fruit fly**. (a) Chart showing the relative occurrences of GO terms for honey bee and fruit fly. Each bar represents the log ratio of the number of proteins annotated with a given category for fly vs. bee. Black bars represent observed proteins in PeptideAtlas; Grey bars represent all annotated proteins. (b) Pie charts representing protein (I, II) and gene (III, IV) coverage in the Honey Bee (I, III) and Fly (II, IV) PeptideAtlases.

Based on these assignments, we then compared the most-commonly matched GO terms between the bee and fruit fly - their whole proteomes and just the non-redundant sequences represented in the respective PeptideAtlases (Figure 5a). Interestingly, a few striking observations emerge from this comparison: e.g., flies seem to have a much larger repertoire of proteins involved in redox metabolism, perhaps reflecting their need to survive in a wider variety of environments and on more diverse food sources than honey bees. On the other hand, ribosomal and other ribonucleoproteins seem to be proportionally more abundant in bees. However this enrichment likely reflects the much greater representation of individual tissues in the bee PeptideAtlas, which contains MS data from systematic dissections of the various body parts. This would be virtually impossible in the much smaller fruit fly.

A comparison of overall gene and protein coverage in the Fly and Honey Bee PeptideAtlases is also illuminating and likewise suggests that greater coverage of various tissues, life stages and castes in honey bee has enabled wider coverage of closely related proteins. Coverage of 'possibly distinguished' and 'subsumed' genes (Figures 5bi and 5bii) proteins (Figure 5biii and 5biv) relative to canonical proteins is higher in bees, as one might expect from greater diversity of samples analyzed. The overall fraction of bee proteins represented in PeptideAtlas is significantly less than for fly partly because there is simply more data available for *Drosophila *but also because we have chosen to use a much larger, more inclusive protein library for Honey Bee than the NCBI library, which contains 9,759 proteins (at the time of writing). While a significant fraction of the 24,558 proteins in the library used in PeptideAtlas are likely not real, we have taken this approach since MS data can provide evidence for proteins that would otherwise be excluded.

### Correction and identification of new proteins

**Figure 6:**
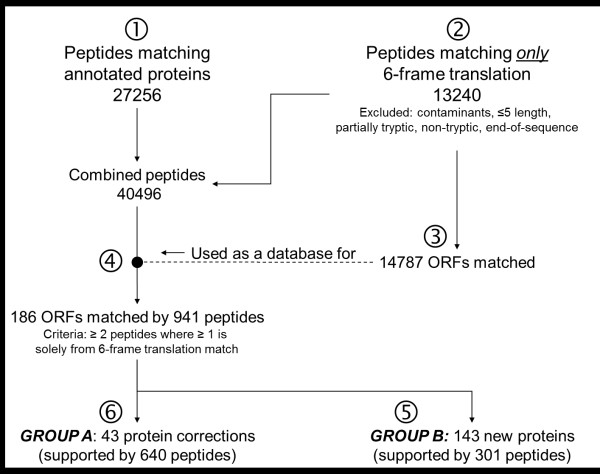
**Annotation and reannotation of bee genes**. MS^2 ^data were searched against a 6-frame translation of all ORFs from the honey bee genome, ignoring matches to contaminants, partial/non-tryptic, short (≤ 5 residues), and end-of-sequence peptides. Peptides that match already annotated proteins are in ➀, and those that did not are in ➁. In ➂, the ORFs matched by ➁ were compiled. In ➃, we tested which peptides can be matched to the ORFs in ➂. The ORFs which are matched only by peptides of ➁ are novel proteins (➄). The ORFs which are matched by peptides from both ➀ and ➁ are corrections of already annotated proteins (➅); most commonly, these were residues that had previously been falsely classified as introns, where current MS spectra now confirm their expression. "GROUP A" and "GROUP B" proteins refer to corrected and new proteins, respectively (see manuscript text).

Typically only 10 to 30% of the fragment spectra from a shotgun proteomics experiment are matched to a peptide when searched against a database of relevant taxonomic constraint. Many apparently high quality spectra are not matched for a number of reasons, the most common of which is thought to be the omission of some post-translational modifications in the database search parameters. However, incomplete gene annotation is another likely cause of unassigned spectra: if the gene has not been identified or has been mis-annotated then the relevant peptide that could match to the spectra in question might not be present in the database. Even *Saccharomyces cerevisiae *genes are not completely annotated but few unannotated proteins can be found, even with deep proteome coverage [29]; for organisms with relatively short histories in genomic research, such as honey bees, the gene annotation is still quite fluid [30]. Furthermore, sometimes predicted genes or experimentally observed ones (e.g., by expressed sequence tags) are not translated *in silico *and therefore not placed into publically accessible protein databases. Given that MS data is not searched by BLAST against a nucleic acid database but by protein database only, this likely represents a significant source of "missing" proteins - an oversight that we hope to address with this proteogenomics effort. By searching a six-frame translation of the entire bee genome we have previously been able to identify several apparently real proteins expressed from unannotated genes (i.e., ones which are not in protein databases) [12] so here we undertook a more systematic and larger-scale re-annotation of bee proteins using MS/MS data (Figure 6). Spectra matching peptides in the existing protein database (27,256 unique sequences, we call these "pre-existing peptides") were separated from the unmatched spectra, using a Perl script. The latter was searched against the ORFs of a six-frame translation of the honey bee genome, resulting in 13,240 peptides (we call these "new peptides") matching to 14,878 ORFs. All spectra were searched against these ORFs, and after omitting the single hits, 186 ORFs remain (Additional File 1) that can be divided into two groups: "Group A" being matched by both pre-existing and new peptides, i.e. corrections of previously annotated proteins, and "Group B" being matched only be new peptides, i.e. novel proteins. Note that Group A proteins likely include mis-annotations of intron-exon boundaries or, possibly, an exon that was entirely missed previously.

We added both Group A and B proteins into PeptideAtlas, but only sections of the ORF as opposed to its entire length. Apart from the matched peptides, we had no further information to map the precise intron-exon boundaries. As a result, we only included the sequence spanned by the two outer-most peptide matches.

**Figure 7:**
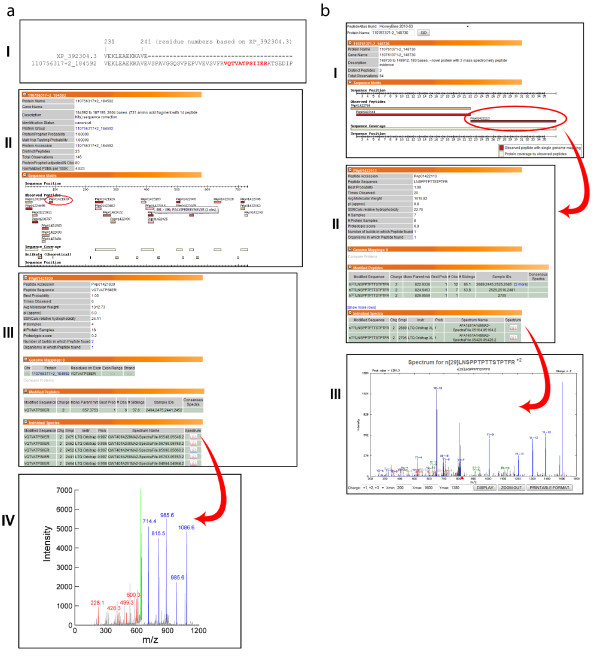
**Example of a corrected protein sequence, using MS-detected peptide as evidence, with screenshots from the PeptideAtlas user interface**. (I) The sequence of XP_392304.3, which is the current version available publicly, is interrupted by an apparent intron after residue 242. However, when searching MS data against a set of ORFs derived from six-frame translations of the bee genome, the peptide VQTVATPSIIER was found only 21 residues C-terminally from residue 242, within what was originally thought to be an intron. (II) Using the PeptideAtlas interface, one can visualize the location of the peptide (circled in red) with respect to the whole protein, (III) explore the frequency of observations of the peptide, and (IV) view the individual spectrum pertaining to each observation. **Figure 7b**. Example of a new protein, using MS-detected peptide as evidence, with screenshots from the PeptideAtlas user interface. (I) The sequence 110761371-2_148730 is an ORF that is not part of the annotated honey bee protein database, yet is matched by three unique peptides. Clicking on any peptide, for example PAp01422113 (circled in red), reveals the sequence itself (LNSPPTPTTSTPTFR - see II), the frequency of observations and more precisely, the samples that contain the peptide spectrum. (III) After clicking on the spectrum icon for any of the samples, the relevant spectrum is shown.

In gene annotation, the most common form of mis-annotation is the incorrect placement of intron-exon boundaries, sometimes assigning a region as an intron where it should be an exon or vice versa. One example of a corrected (Group A) protein is in Figure 7a, where the peptide VQTVATPSIIER (PAp01421939, seven spectral counts) matched a contig (110756317+2_184592), from which protein XP_392304.3 was previously annotated. VQTVATPSIIER falls in the intronic region but the very high quality MS/MS spectra indicates that it is indeed real and that the intron-exon boundaries are mis-assigned for this protein.

Gene annotation relies heavily on automated algorithms and pattern matching, which can sometimes completely miss real genes. Figure 7b shows an example a novel (Group B) protein - an ORF with several high-quality matches from new peptides. A BLAST search against the non-redundant database revealed no significant hits, which is not surprising, given that gene annotation algorithms often rely on sequence similarity against other organisms and so if there had been a hit in another organism, this gene might have been identified as such. It should be noted that since most bee genome contigs have not be scaffolded, it is possible that one protein may span multiple contigs; thus, some of the novel, expressed ORFs detected here may come from the same protein.

In order to facilitate analysis of future honey bee shotgun experiments, we have compiled a spectral library based on all the identifications in the PeptideAtlas build using the SpectraST library building tool [31]. SpectraST collects all replicate spectra for each peptide ion and creates a consensus spectrum based on a voting scheme that retains repeated peaks. This can enhance future experiments because SpectraST searches are many times faster than conventional sequence searching and SpectraST scores can discriminate better between correct and incorrect identifications [32]. The spectrum library in splib and sptxt format is available at the same URL with the other data products from this work.

Targeted proteomics workflows via selected reaction monitoring (SRM) enable highly sensitive and repeatable quantitative measurements on triple-quadrupole mass spectrometers [33]. However, such workflows require considerable experiment planning to select the specific signatures needed to detect the target peptides [34]. To aid in this experiment planning, we have created an SRMAtlas build [35] based on the ion trap observations and predictions. The results of this process are available at the SRMAtlas web site http://www.srmatlas.org.

## Conclusions

We present here the first publicly accessible resource for honey bee proteomics using the PeptideAtlas architecture [7]. In addition to providing the experimental evidence behind each peptide identification, we have also undertaken a proteogenomic re-annotation of honey bee proteins that has led to the identification of 186 new or mis-annotated regions of bee proteins. We expect that as more MS/MS data are collected, we will be able to further refine the annotation of honey bee genes.

Honey bees are typically studied to reveal the biological underpinnings of a complex insect society and rarely as a model of human disease or basic biology. Consequently, there is a dearth of detection reagents and probes for honey bee proteins. As we and others move into using selected reaction monitoring as a means for targeting specific proteins, the MS/MS data presented in the Honey Bee PeptideAtlas will provide the empirical evidence required to make intelligent decisions about the design of these experiments. The importance of honey bee populations to natural and agro-ecological systems, coupled to their current decline obligates us to improve the status quo. The release of these data into the public domain not only represents validation and improvement of the current annotation of the reference genome, but provides the empirical evidence to guide future honey bee biochemical research.

## Availability and Requirements

• **Project name: **HoneyBee PeptideAtlas

• **Project home page: **https://db.systemsbiology.net/sbeams/cgi/PeptideAtlas/buildDetails?atlas_build_id=282

• **Operating system(s): **e.g. Platform independent

• **Programming language: **N/A

• **Other requirements: **none

• **License: **Creative Commons Attribution

• **Any restrictions to use by non-academics: **none

## Abbreviations

BLAST: Basic Local Alignment Search Tool; EST: expressed sequence tags; FDR: false discovery rate; GO: Gene Ontology; HBPA: Honey Bee PeptideAtlas; HSP: high-scoring segment pair; MS: mass spectrometry; MS/MS: tandem mass spectrometry; ORF: open reading frame; SRM: single reaction monitoring; STAGE Tips: STop-And-Go Extraction tips

## Authors' contributions

EWD and LJF conceived of the project idea. QWTC compiled the mass spectra for this study. QWTC did the proteogenomics analysis with help from RP. ZS processed the raw data to generate the Honey Bee PeptideAtlas. QWTC, EWD and LJF wrote the initial version of the manuscript. All authors have read and approved the final manuscript.

## Supplementary Material

Additional file 1 **Correct or novel honey bee proteins supported by MS-based peptide spectra**. Peptide spectra were searched against six frame-translated honey bee ORFs to augment currently existing, publically available protein databases. Each protein in this file is hyperlinked to the relevant entry in PeptideAtlas, which provides a graphical display of MS-based peptide evidence for each Corrected (Column A) or Novel Protein (Column B). Click here for file
